# Supplementary material for: Uncovering nitroxoline activity spectrum, mode of action and resistance across Gram-negative bacteria
Source: Nat Commun. 2025 Apr 22;16:3783. doi: 10.1038/s41467-025-58730-5 (PMC12015411; doi:10.1038/s41467-025-58730-5)
Supplement: Supplementary file 1 — Supplementary Information [file 41467_2025_58730_MOESM1_ESM.pdf]

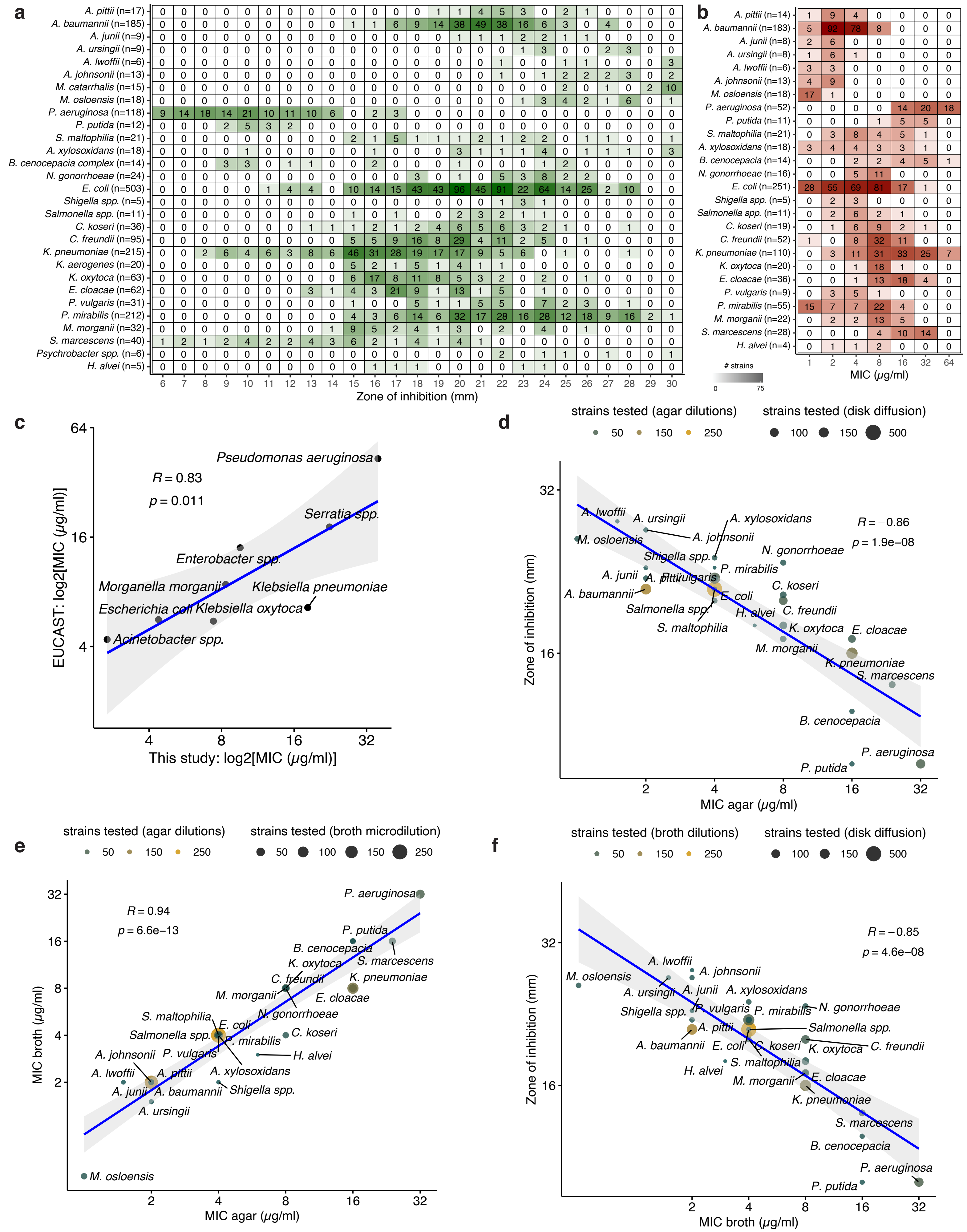

Supplementary Fig. 1. Nitroxoline MIC measurement via disk diffusion and agar dilutions and agreement across the different assays and with EUCAST. a-b. MIC measurements via disk diffusion (**a**) and agar dilution (**b**). Sensitivity is expressed as inhibition zone diameter (mm) (**a**) or μg/ml (**b**) (Methods). Each value is annotated and color-coded according to the number of strains it was measured in. Multiple species are aggregated for *Salmonella spp.*, *Shigella spp.*, *Burkholderia cenocepacia complex* and *Psychrobacter spp.* (Source Data). **c.** Concordance between MICs measured in broth dilution in this study and according to EUCAST[19] for the eight overlapping species. **d-f.** Concordance between MICs measured across three orthogonal methods: agar dilution and disk diffusion (**d**), broth and agar dilution (**e**), broth dilution and disk diffusion (**f**). Pearson's correlation coefficient and p-value are shown. Disk diffusion results are expressed as in Supplementary Fig. 1a.

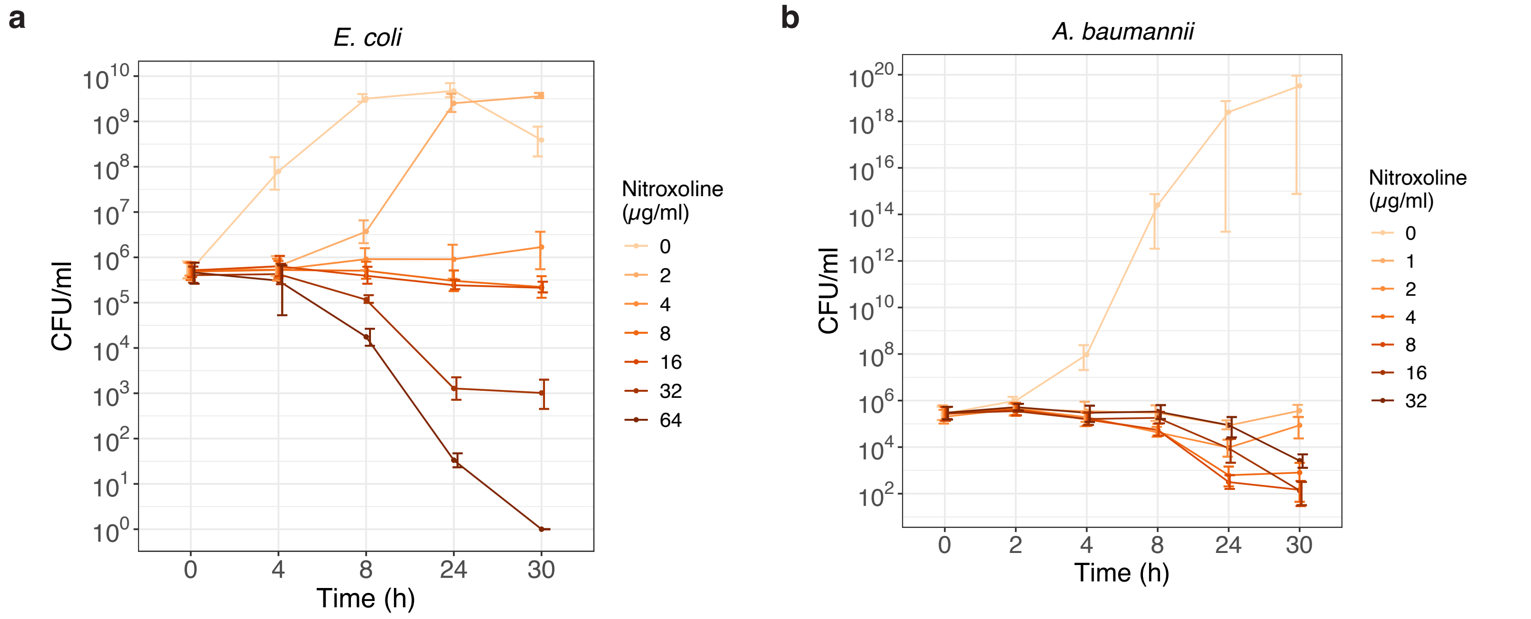

Supplementary Fig. 2. Bactericidal activity of nitroxoline. **a.** Nitroxoline is bacteriostatic against *E. coli* BW25113 at concentrations up to 4 x MIC (16 µg/ml) and bactericidal from 8 x MIC (32 µg/ml). Results are represented as in Fig. 1e. **b.** Time-kill curves for nitroxoline in *A. baumannii* ATCC 19606T (Fig. 1e) including the no-drug control. Results are represented as in Fig. 1e.

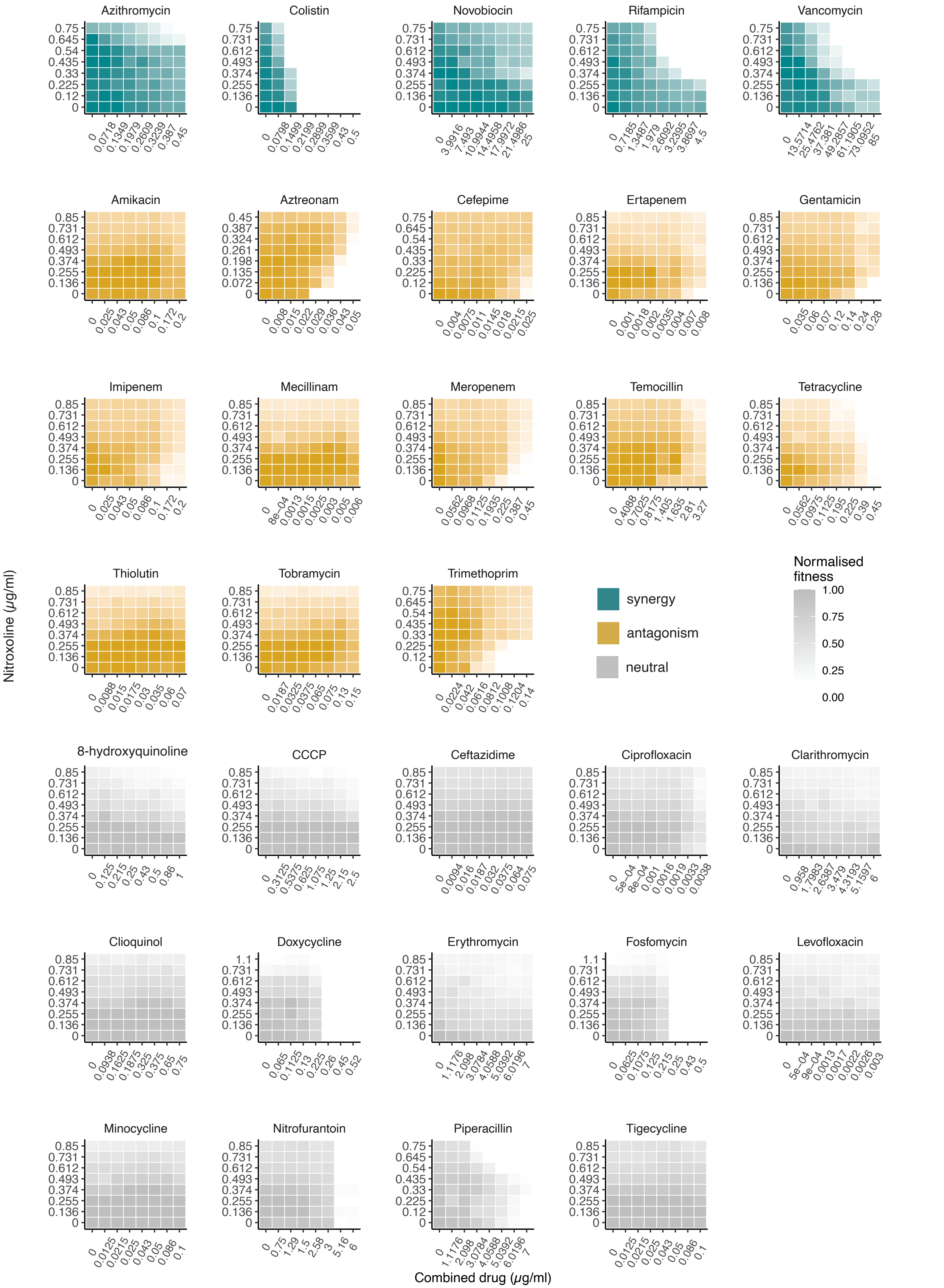

Supplementary Fig. 3. Checkerboard microdilution of nitroxoline combinations with 32 antimicrobials. Drugs were tested in combination with nitroxoline in 8 x 8 broth microdilution checkerboards in *E. coli* BW25113. The median fitness ($OD_{595\ nm}$ at 7.5 h normalized by no-drug controls) across at least two biological replicates is shown (distinct replicates available at https://github.com/ElisabettaCacace/nitroxoline_2024/blob/main/figures/additional_figures.pdf).

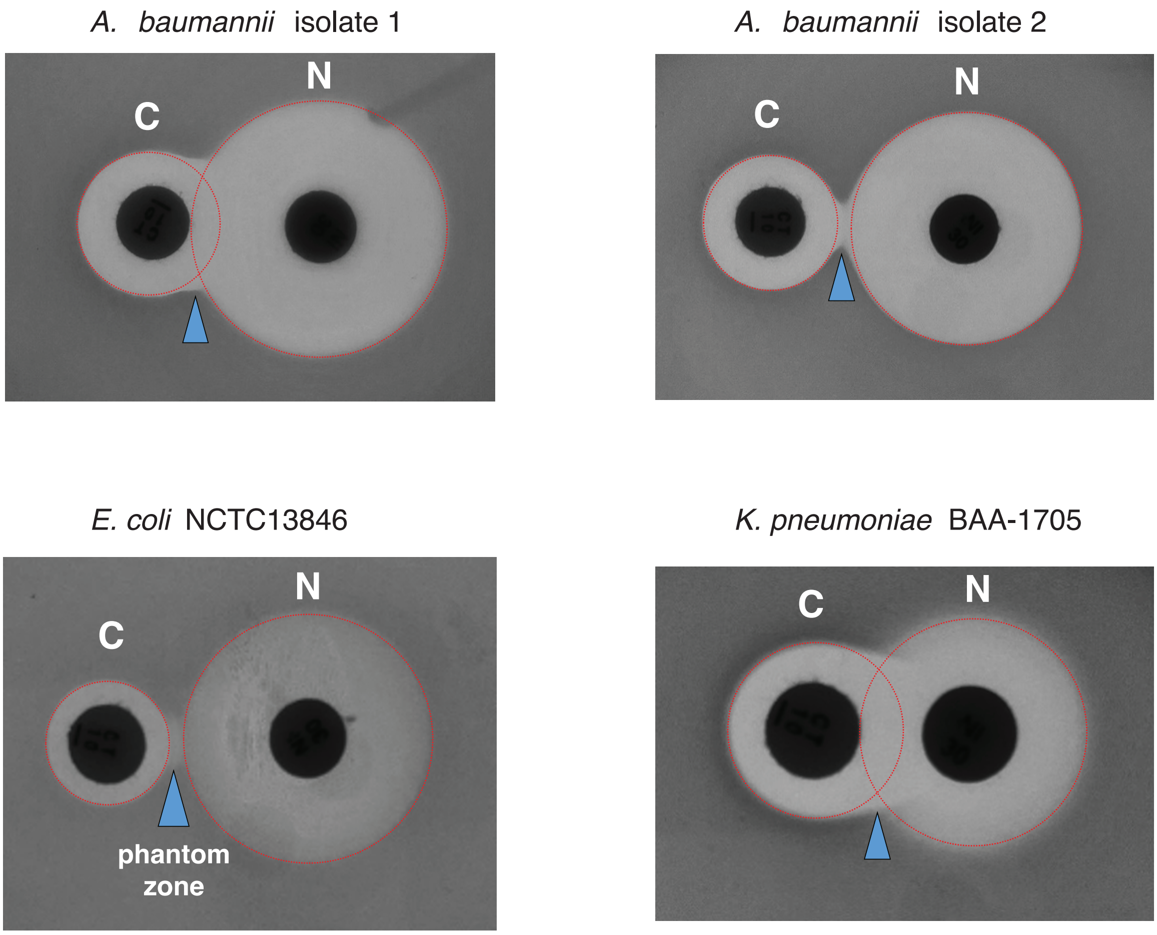

Supplementary Fig. 4. Colistin-nitroxoline synergy across Gram-negative species (disk diffusion). A nitroxoline disk (N; 30 μg, Liofilchem) and a colistin disk (C; μg, Oxoid) were placed on a Mueller-Hinton agar plate (Oxoid), inoculated with the indicated isolate. The red dotted line indicates the zone of inhibition (ZOI), the blue arrow the synergism as widened circular ZOI or additional growth inhibition outside the ZOI (phantom zone). Interactions are evident despite the large molecular weight of colistin, which prevents effective diffusion into the agar.

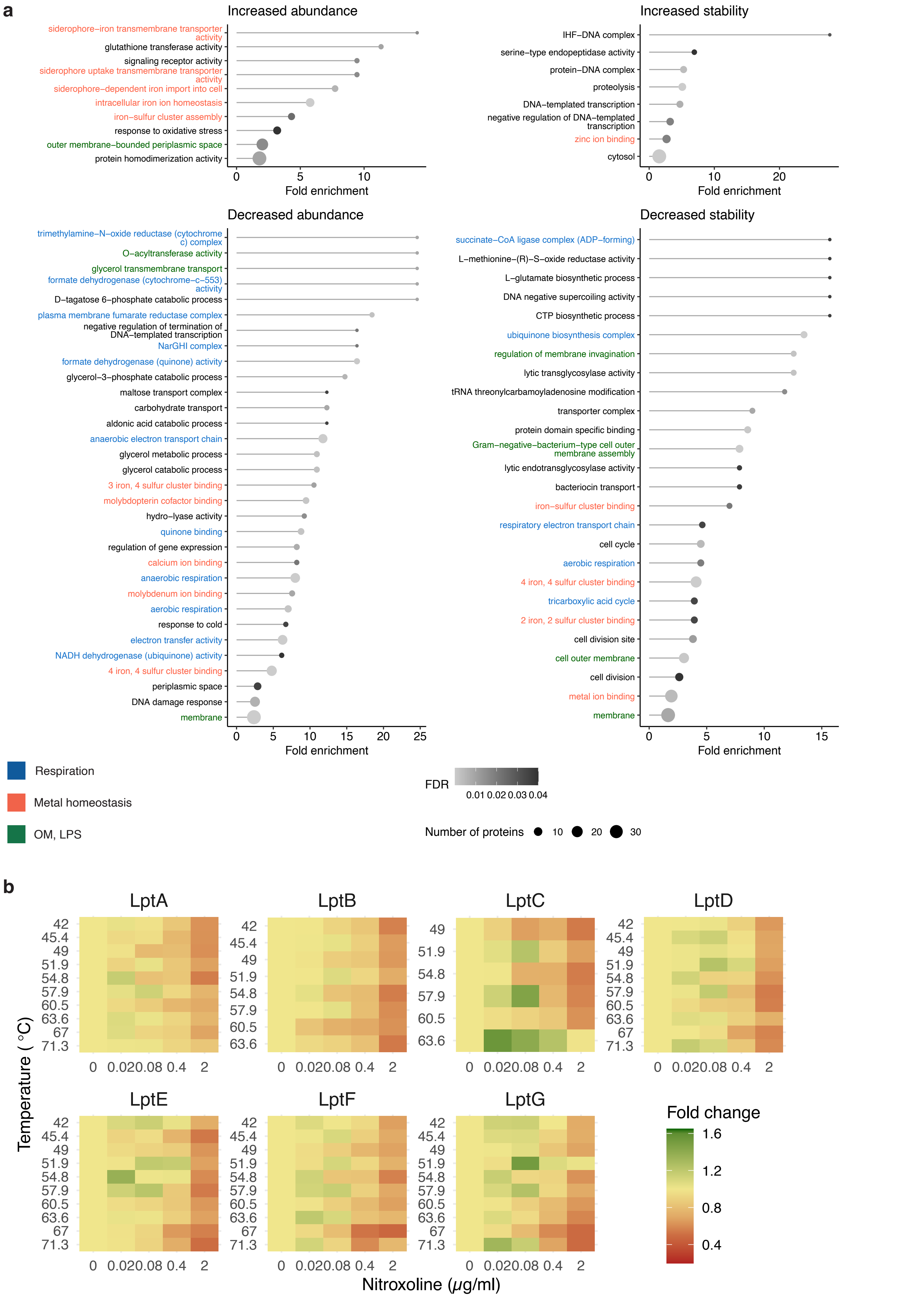
a
Increased abundance
siderophore–iron transmembrane transporter activity
glutathione transferase activity
signaling receptor activity
siderophore uptake transmembrane transporter activity
siderophore–dependent iron import into cell
intracellular iron ion homeostasis
iron–sulfur cluster assembly
response to oxidative stress
outer membrane–bounded periplasmic space
protein homodimerization activity
Fold enrichment
Increased stability
IHF–DNA complex
serine–type endopeptidase activity
protein–DNA complex
proteolysis
DNA–templated transcription
negative regulation of DNA–templated transcription
zinc ion binding
cytosol
Fold enrichment
Decreased abundance
trimethylamine–N–oxide reductase (cytochrome c) complex
O–acyltransferase activity
glycerol transmembrane transport
formate dehydrogenase (cytochrome–c–553) activity
D–tagatose 6–phosphate catabolic process
plasma membrane fumarate reductase complex
negative regulation of termination of DNA–templated transcription
NarGHI complex
formate dehydrogenase (quinone) activity
glycerol–3–phosphate catabolic process
maltose transport complex
carbohydrate transport
aldonic acid catabolic process
anaerobic electron transport chain
glycerol metabolic process
glycerol catabolic process
3 iron, 4 sulfur cluster binding
molybdopterin cofactor binding
hydro–lyase activity
quinone binding
regulation of gene expression
calcium ion binding
anaerobic respiration
molybdenum ion binding
aerobic respiration
response to cold
electron transfer activity
NADH dehydrogenase (ubiquinone) activity
4 iron, 4 sulfur cluster binding
periplasmic space
DNA damage response
membrane
Fold enrichment
Decreased stability
succinate–CoA ligase complex (ADP–forming)
L–methionine–(R)–S–oxide reductase activity
L–glutamate biosynthetic process
DNA negative supercoiling activity
CTP biosynthetic process
ubiquinone biosynthesis complex
regulation of membrane invagination
lytic transglycosylase activity
tRNA threonylcarbamoyladenosine modification
transporter complex
protein domain specific binding
Gram–negative–bacterium–type cell outer membrane assembly
lytic endotransglycosylase activity
bacteriocin transport
iron–sulfur cluster binding
respiratory electron transport chain
cell cycle
aerobic respiration
4 iron, 4 sulfur cluster binding
tricarboxylic acid cycle
2 iron, 2 sulfur cluster binding
cell division site
cell outer membrane
cell division
metal ion binding
membrane
Fold enrichment
Respiration
Metal homeostasis
OM, LPS
FDR
0.01 0.02 0.03 0.04
Number of proteins 10 20 30
b
LptA
LptB
LptC
LptD
LptE
LptF
LptG
Temperature (°C)
Nitroxoline (μg/ml)
0 0.02 0.08 0.4 2
Fold change
1.6
1.2
0.8
0.4

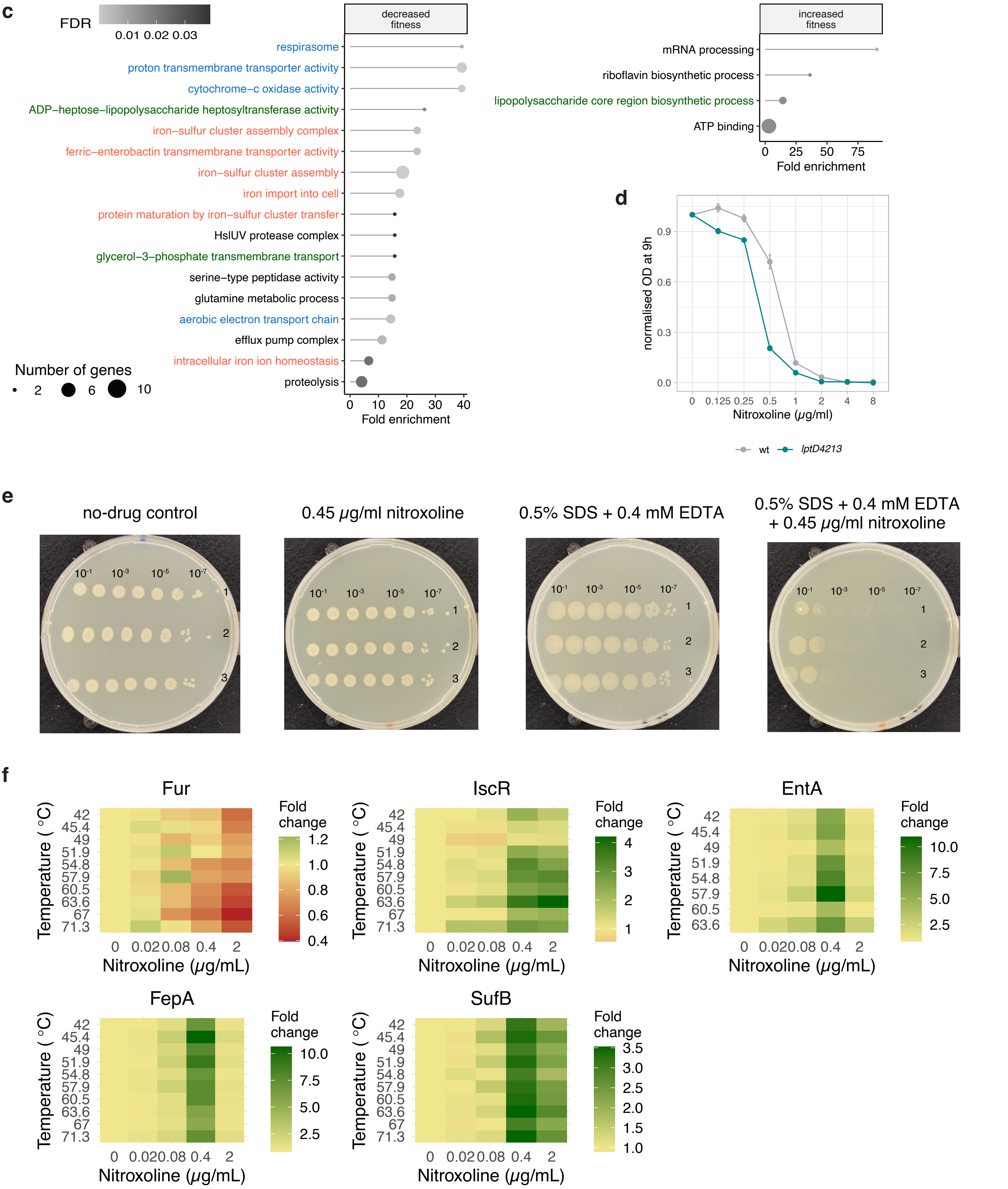

Supplementary Fig. 5 (continued). Nitroxoline perturbs the OM and affects metal homeostasis in *E. coli*. a. GO enrichment of 2D-TPP abundance and stability changes (Fig. 3a, Supplementary Data 3). Significantly enriched GO terms are shown (adjusted p-value < 0.05, Fisher's exact test). Dot size represents the number of hit proteins for each term, dot colour the adjusted p-value, label colour highlights GO terms related to OM or LPS, metal homeostasis, and respiration. **b.** Nitroxoline affects the abundance and stability of the LPS transport machinery. In the thermal stability profiles of Lpt system members, protein fold change is shown for each temperature and nitroxoline concentration. **c.** GO enrichment of chemical genetics hits (Fig. 3b, Supplementary Data 4). Results are represented as in Supplementary Fig. 5a. **d.** Nitroxoline is more potent upon genetic perturbation of the OM. Nitroxoline susceptibility in *E. coli* BW25113 and its OM-defective derivative strain, carrying the *lptD4213* mutation[44]. Data is shown as in Fig. 2b. For full growth curves see https://github.com/ElisabettaCacace/nitroxoline_2024/blob/main/figures/additional_figures.pdf. **e.** Lower concentrations of nitroxoline and EDTA were tested than in Fig. 3d (EOP assay, Methods) to show the first dosage at which growth was visible upon combination. **f.** Nitroxoline induces effects in 2D-TPP consistent with iron-sulfur cluster disruption. Thermal profiles are represented as in Supplementary Fig. 5b.

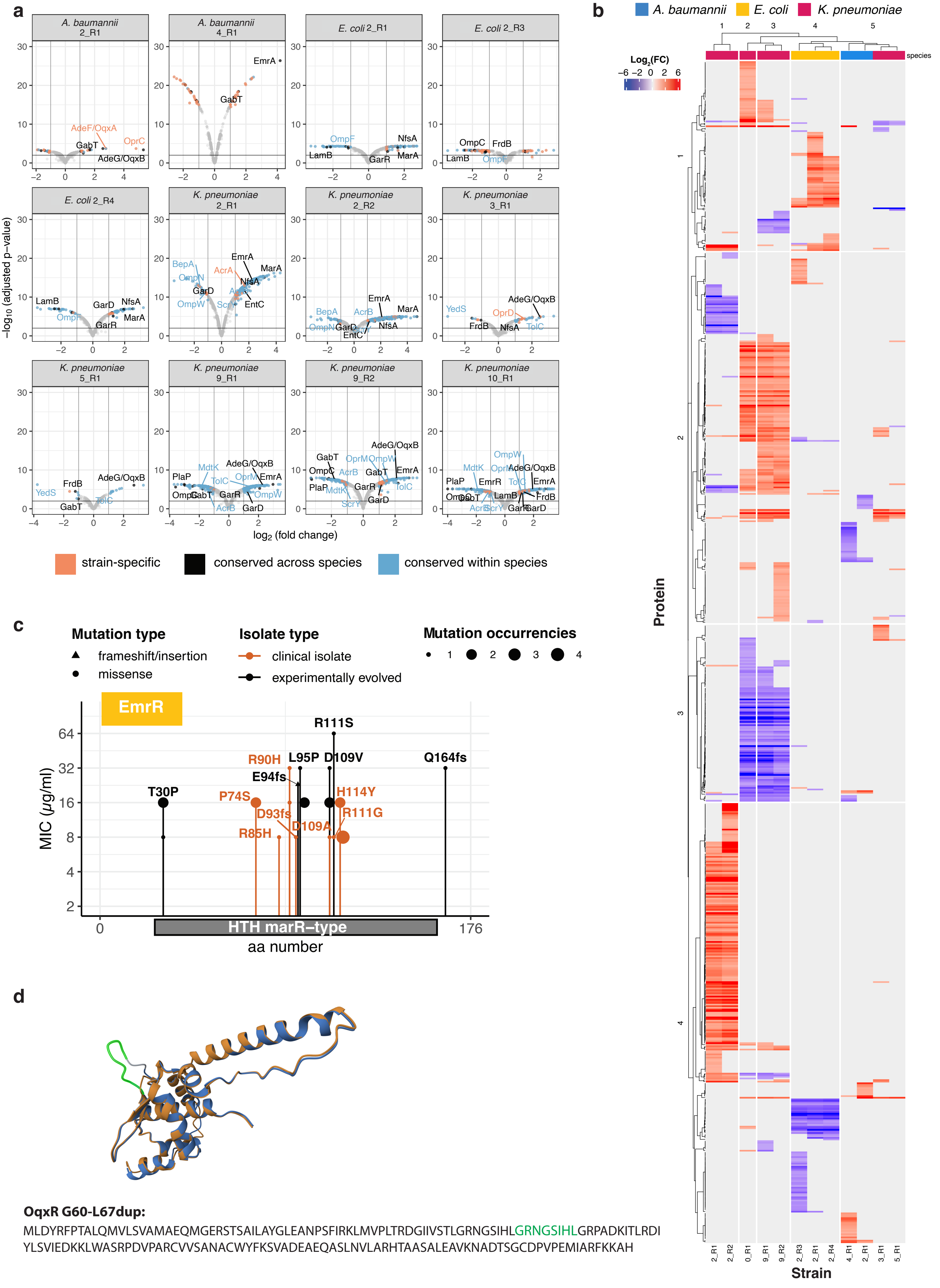

a
A. baumannii 2_R1
A. baumannii 4_R1
E. coli 2_R1
E. coli 2_R3
E. coli 2_R4
K. pneumoniae 2_R1
K. pneumoniae 2_R2
K. pneumoniae 3_R1
K. pneumoniae 5_R1
K. pneumoniae 9_R1
K. pneumoniae 9_R2
K. pneumoniae 10_R1
−log10 (adjusted p−value)
log2 (fold change)
strain-specific
conserved across species
conserved within species
b
A. baumannii
E. coli
K. pneumoniae
Log2(FC)
species
Protein
Strain
c
Mutation type
frameshift/insertion
missense
Isolate type
clinical isolate
experimentally evolved
Mutation occurrencies
EmrR
MIC (μg/ml)
HTH marR−type
aa number
d
OqxR G60-L67dup:
MLDYRFPTALQMVLSVAMAEQMGERSTSAILAYGLEANPSFIRKLMVPLTRDGIIVSTLGRNGSIHLGRNGSIHLGRPADKITLRDI
YLSVIEDKKLWASRPDVPARCVVSANACWYFKSVADEAEQASLNVLARHTAASALEAVKNADTSGCDPVPEMIARFKKAH

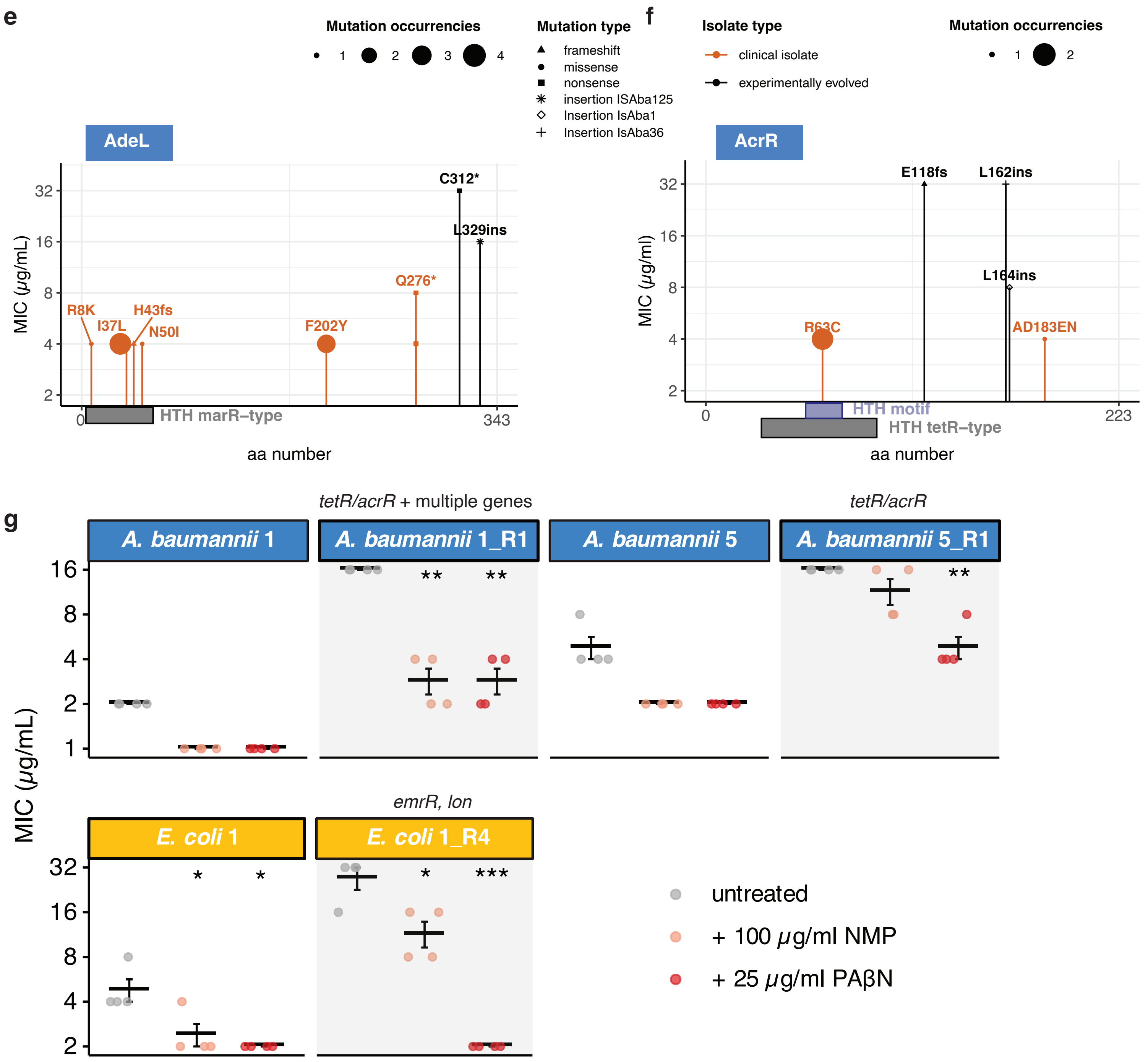

Supplementary Fig. 6. Nitroxoline resistance is based on conserved mechanisms across species. **a.** Volcano plots of abundance changes in proteomics of resistant strains compared to isogenic sensitive strains. Effect size and statistical significance (Methods) are represented on the x- and y- axis, respectively. Data points are colour-coded according to their conservation across or within-species. For sensitive strains see https://github.com/ElisabettaCacace/nitroxoline_2024/blob/main/figures/additional_figures.pdf. **b.** Protein abundance changes in nitroxoline-resistant strains. Only significant and strong changes (adjusted p-value ≤ 0.01, $|\log_2$ fold-change (abundance)$| \geq 2$) are included (Supplementary Data 6). Proteins and strains are clustered according to Pearson's correlation. Results are represented as in Fig. 5b. **c.** Amino acid changes from *emrR* mutations in clinical and experimentally evolved nitroxoline-resistant isolates. Results are represented as in Fig. 5d. **d.** Superimposition of mutated and wild-type OqxR structure. The G60-L67 duplication loop is highlighted in green in the structure (Methods) and in the OqxR amino acid sequence below. **e-f.** Amino acid changes from *adeL* (**e**) and *acrR/tetR* (**f**) mutations in clinical and experimentally evolved nitroxoline-resistant isolates. Results are represented as in Fig. 5d. **g.** Efflux-pump inhibitors resensitize nitroxoline-resistant strains. Results are represented as in Fig. 5e.

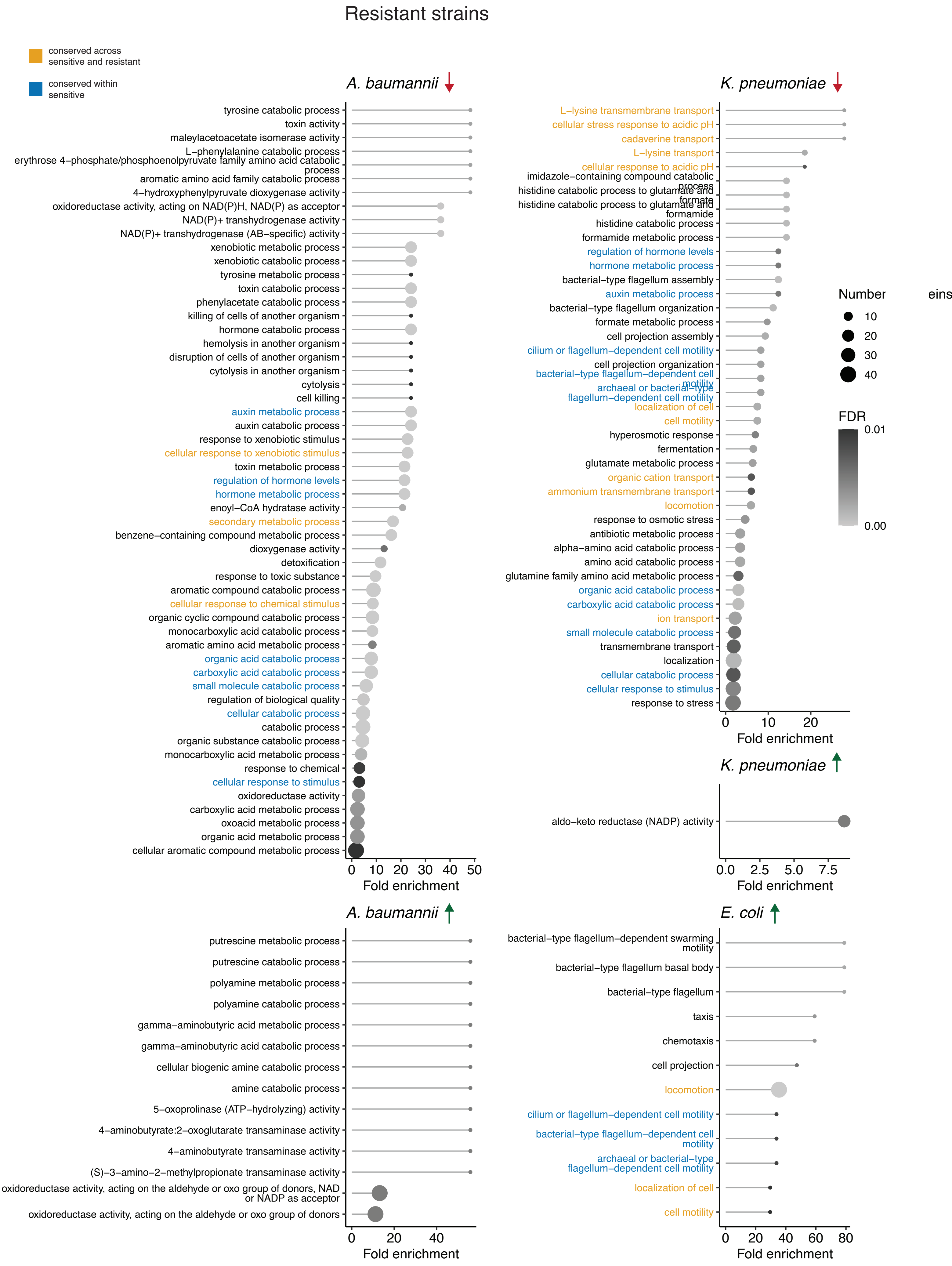

Supplementary Fig. 7. GO enrichment of significant hits from proteomics on nitroxoline-resistant strains (Fig. 5b, Supplementary Fig. 6b, Supplementary Data 6). Only sets yielding significant enrichments (down- or up-regulation in the indicated species) are shown (adjusted p-value < 0.05, one-sided Fisher's exact test). The number of protein hits is annotated for each term).

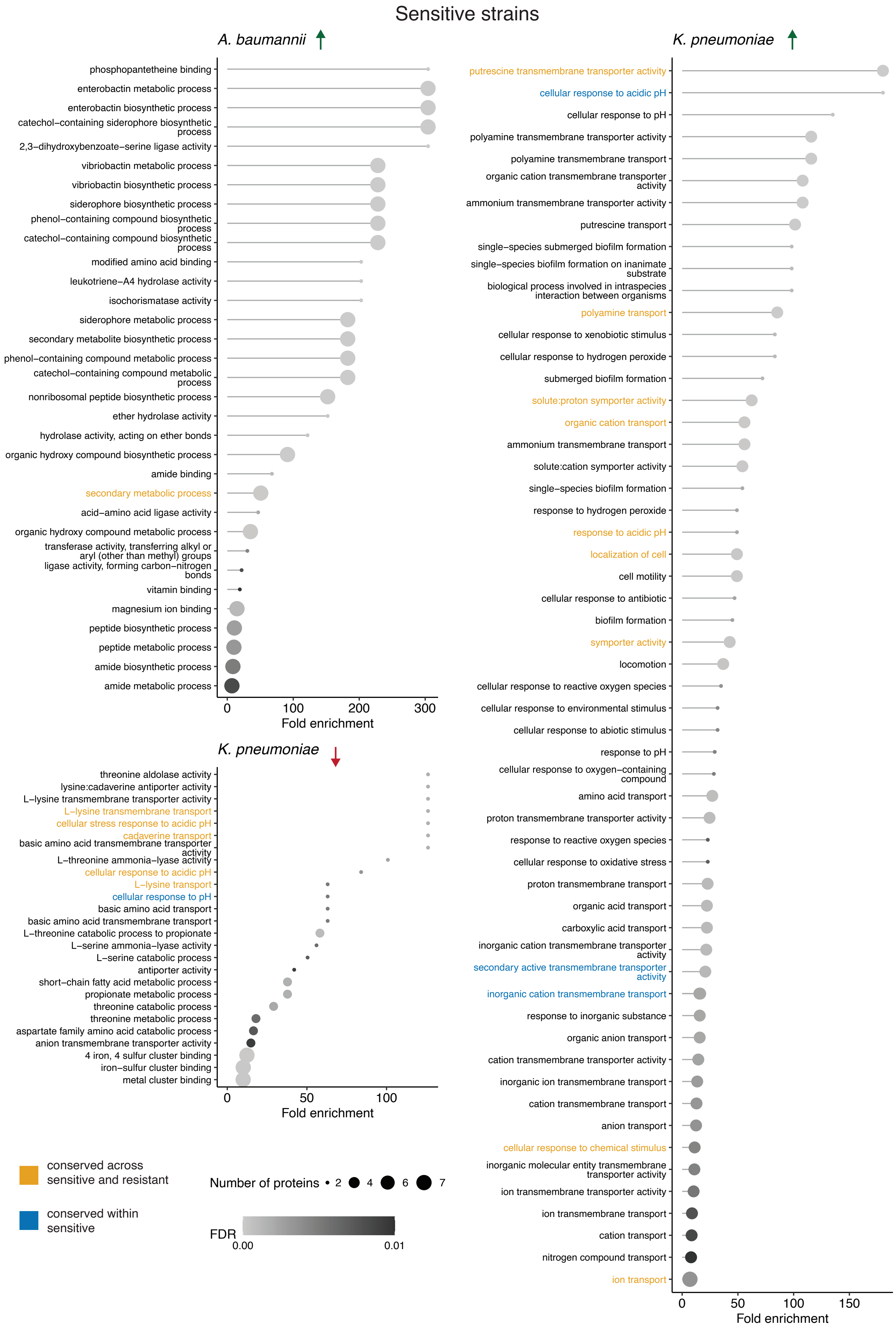

Supplementary Fig. 8. GO enrichment of significant hits from proteomics on nitroxoline-sensitive strains. Data is represented as in Supplementary Fig. 7.

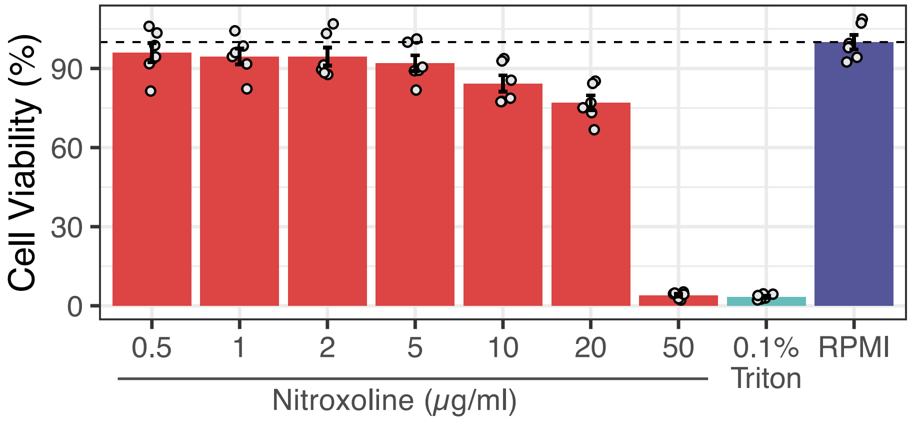

Supplementary Fig. 9. MTT cell viability assay on HeLa cells exposed to increasing concentrations of nitroxoline. Controls include 0.1% Triton and RPMI as positive and negative controls, respectively. Results are expressed as mean and standard error across six biological replicates. 5 μg/ml is the nitroxoline dose used for the intracellular *S.*Typhi killing assay shown in Fig. 1d.
