# Supplementary material for: Uncovering nitroxoline activity spectrum, mode of action and resistance across Gram-negative bacteria
Source: Nat Commun. 2025 Apr 22;16:3783. doi: 10.1038/s41467-025-58730-5 (PMC12015411; doi:10.1038/s41467-025-58730-5)
Supplement: Supplementary file 2 — Description of Additional Supplementary Files [file 41467_2025_58730_MOESM2_ESM.pdf]

Description of Additional Supplementary Files:

Supplementary Data 1: The drugs tested in combination with nitroxoline in E. coli BW25113, their chemical class and highest concentration tested.

Supplementary Data 2: All the strains used in this study, their genotype, antibiotic resistance and nitroxoline MIC, except for those shown in Fig. 1b-c and Supplementary Fig. 1a-b (see Source Data).

Supplementary Data 3: 2D-TPP data from *E. coli* BW25113 wholecell samples exposed to nitroxoline (Methods).

Supplementary Data 4: Mutant fitness as log(fold change) and FDR from the chemical genetic screen performed on the E. coli Keio collection with nitroxoline (Methods).

Supplementary Data 5: Frequency of nitroxoline resistance in *A. baumannii*, *E. coli*, *K. pneumoniae*.

Supplementary Data 6: Proteomics data from nitroxoline-sensitive and resistant strains listed in Supplementary Data 1 and annotated with their orthologous groups (Methods).

Supplementary Movie 1: Time-lapse of A. baumannii growing on cation-adjusted Mueller-Hinton-agarose 1% pad, supplemented with 8 μg/ml nitroxoline.

Supplementary Movie 2: Time-lapse of A. baumannii growing on cation-adjusted Mueller-Hinton-agarose 1% pad, not supplemented with 8 μg/ml nitroxoline.

Supplementary Movie 3: Time-lapse of E. coli growing on cationadjusted Mueller-Hinton-agarose 1% pad, supplemented with 2 μg/ml nitroxoline.
